# Effect of aged garlic powder on physicochemical characteristics, texture profiles, and oxidative stability of ready-to-eat pork patties

**DOI:** 10.5713/ajas.18.0689

**Published:** 2019-01-03

**Authors:** Ji-Han Kim, Hyun-Joo Jang, Chi-Ho Lee

**Affiliations:** 1 Department of Food and Bioproduct Sciences, University of Saskatchewan, Saskatoon, SK, S7N5A8, Canada; 2 Department of Food Science & Biotechnology of Animal Resources, Konkuk University, Seoul 05029, Korea

**Keywords:** Aged Garlic, Patties, Thiol, Ready-to-eat, Lipid Oxidation

## Abstract

**Objective:**

The aim of this study was to investigate the effects of aged garlic powder (AGP) on physicochemical characteristics, texture profiles, and oxidative stability of ready-to-eat (RTE) pork patties.

**Methods:**

There were five treatment groups: a control; 1% fresh garlic powder (T1); 0.5%, 1%, and 2% AGP (T2, T3, and T4). Pork patties with vacuum packaging were roasted at 71°C for core temperature, stored at 4°C for 14 d, and then reheated for 1 min using a microwave.

**Results:**

The AGP groups showed a lower the level of lipid oxidation and higher thiol contents than the control and T1. The pH value of the control increased whereas that of aged garlic groups decreased after re-heating process. In addition, the redness significantly increased with increasing level of AGP whereas the redness of the control and T1 decreased after re-heating process. T4 added patties improved textural and sensory properties compared to the control.

**Conclusion:**

The results of this study suggest that AGP addition to RTE pork patties can improve their sensory characteristics and oxidative stability.

## INTRODUCTION

In recent years, garlics have been used as a main ingredient for meat product. Garlics provide plentiful phenol compounds and sulfur-containing compounds such as cysteine, alliin, and allyl disulfide to improve flavor and extent shelf-life of meat products [1–3]. In addition, heating process on garlic significantly improves taste-active compounds, which are related to strong mouthfullness (kokumi taste), through Maillard reaction [4]. The Maillard reaction between amino acids and glucose contributes to the occurrence of non-enzymatic browning reaction that develops color and flavor intensity during cooking [3].

Aged garlic (AG) is well-known as black garlic which is processed by heat (50°C to 90°C) and time (more than one month) treatments [5]. Antioxidant ability of AG is increased compared to fresh garlic (FG) since S-allyl cysteine of AG is increased with Maillard reaction during such processing [6]. In terms of human/animal diet, functionalities such as anti-allergic and antioxidant activities of AG have been determined in previous studies [7,8]. Recently, the application of AG in foods such as sausage [9] and sponge cake [10] has been studied, revealing that AG can extend shelf life of foods.

Recently, consumers prefer to eat food with more convenience, resulting in the growth of ready-to eat (RTE) food market [11]. Many studies have been performed to inhibit lipid and protein oxidation of RTE products [12–14]. Liberation of heme iron from myoglobin during cooking and reheating accelerates oxidation of RTE meat [15]. Meat deterioration is mainly derived from lipid and protein oxidation with formation of volatile compounds responsible for off-flavors and rancid odor [16].

Based on these reports, we hypothesized that the addition of AG to ready to eat pork patties could positively influence their sensory characteristics and oxidative stability. Therefore, the aim of this study was to investigate the effect of AG on quality and oxidative stability of RTE pork patties.

## MATERIALS AND METHODS

### Preparation of sample and pork patties

**Figure 1:**
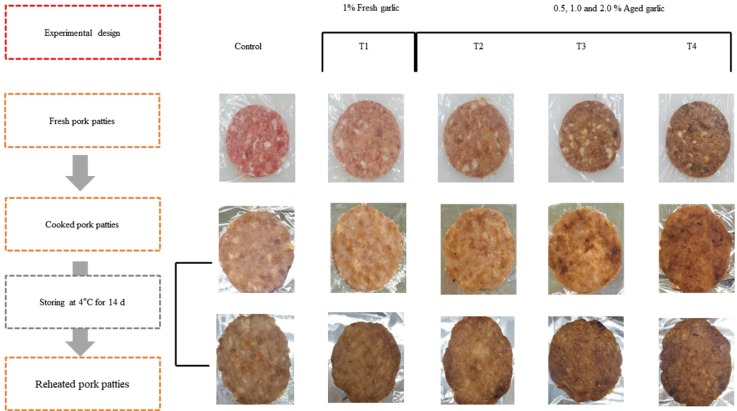
Schematic figure showing experimental design of this study. Control, pork patties without fresh or aged garlic powder; T1, 1% fresh garlic (w/w), T2, T3, and T4, 0.5%, 1%, and 2% aged garlic (w/w).

The FG and AG powders were obtained from OZL DNF Inc. (Dam Yang, Korea). Garlic samples (FG and AG) were freeze-dried for powdering process. Fresh pork hams (lean meat) and back fat were obtained from GUMDON Corp. (Won Ju, Korea). Pork patties (65 g) were processed by finger kneading at once using patty-maker (small round burger press, Spikomat Ltd., UK) into approximately 100 mm diameter and 10 mm thickness with the basic formulations given in lean meat 785 g/kg, back fat 200 g/kg and sodium chloride 15 g/kg (Control group, without garlic powder; T1, 1% FG powder; T2, T3, and T4, 0.5%, 1%, and 2%, respectively). Each of the five treatments was performed with three different manufacturing processes as follows (Figure 1): i) For fresh patties, color, pH, and proximate compositions of raw patties were measured immediately after production; ii) the pork patties were grilled in open electric griddles (Preheated temperature 260°C±5°C, HANSOL Inc., Seoul, Korea). Patties were cooked on the first side for 1 min, the second side for 1 min, and then each side turned for a final 2 min for a total of 6 min according to the method of Berry and Bigner-George [17]; iii) For reheated patties (RTE), pre-cooked patties were refrigerated at 4°C for 14 d in a polyethylene/nylon vacuum bags (Cryovac, Duncan, SC, USA). They were then cooked with a microwave. The microwave oven (RE-338BR, Samsung Electronics Co., Ltd., Suwon, Korea) was pre-set to 500 mW for 1 min. After cooking, patties were allowed cool off at room temperature for experimentation. All experiments were performed in triplicates.

### Proximate compositions analysis

Proximate compositions (moisture, crude protein, crude fat, and ash) were analyzed using methods of the Association of Official Analytical Chemists [18].

### Color measurement

Color of samples was measured using a colorimeter (CR-400, Konica Minolta Sensing, Inc., Osaka, Japan). The colorimeter was calibrated with standard (Commission Internationale de l’Eclairage [CIE], L* = +97.43, CIE a* = −0.05, CIE b* = +1.92). CIE L*-value (lightness), CIE a*-value (redness), and CIE b*-value (yellowness) were determined for the surface of each sample.

### Browning intensity of Maillard reaction products

Brown intensity (BI) of Maillard reaction products (MRPs) was determined for cooked and reheated pork patties by the method of Smith and Alfawaz [19]. Briefly, 2 g of sample was homogenized with 18 mL of distilled water and then filtered through Whatman paper No. 1. The BI of sample was measured at wavelength of 420 nm using a spectrophotometer (Libra S22, Biochrom, Cambridge, England). The BI difference was calculated as follows: the ratio of cooked sample to fresh sample (ΔC) and the ratio of reheated sample to fresh sample (ΔR).

### pH measurement

The pH value of sample was measured with a digital pH-meter (LAQUA F-71, HORIBA, Kyoto, Japan). Briefly, 2 g of sample and 18 mL of distilled water were added into a bag filter. The sample was blended using a Bag Mixer (400 P, Interscience Co, Saint Nom, France) for 90 s followed by pH measurement.

### Cooking loss

After cooking, patties were cooled at room temperature. Cooking loss was computed using the following equation:

$$\text{Cooking loss }(\%)=\frac{\left[\text{Weight of patty before cooking}(\text{g})-\text{Weight of patty after cooking}(\text{g})\right]}{\text{Weight of patty before cooking}(\text{g})}\times 100$$

### 2-Thiobarbituric acid reactive substances

Lipid oxidation was determined in triplicates using 2-Thiobarbituric acid reactive substances (TBARS) method of Tarladgis et al [20] with slight modifications. Briefly, 5 g sample was homogenized with 25 mL distilled water for 1 min at 10,000 rpm. The mixture was then transferred to a distillation flask. The tube used for mixing was washed with an additional 23.5 mL of distilled water. To the same distillation flask, 4 N HCl (1.25 mL) with antifoam agent (1 mL) a few boiling stones were added to prevent puffing. The mixture was heated to obtain distillate. Heating was stopped when 30 mL of distillate was collected in the other tube. To each 5 mL of collected distillate, 5 mL of TBA reagent (0.002 M 2-thiobarbituric acid in 90% acetate) was added and well-mixed. The mixture was then heated in water bath at 100°C for 30 min to develop chromogen followed by cooling in a refrigerator. Absorbances were measured at wavelength of 538 nm on a UV/vis spectrophotometer (Libra S22, Biochrom, England) and calculated as mg malondialdehyde (MDA)/kg meat using 1,1,3,3-tetraethoxypropane to prepare a standard curve.

### Determination of sulfhydryl groups

Total sulfhydryl (thiol) content was quantified spectrophotometrically after derivatization by Ellman’s reagent 5,5′-Dithiobis (2-nitrobenzoic acid) (DTNB) as described by Jongberg et al [21]. Briefly, 1 g of patty sample was homogenized in 20 mL of 5% sodium dodecyl sulfate (SDS) buffer for 30 s. Homogenized sample was placed in a water bath at 80°C for 1 h followed by cooling. The solution was filtered through a Whatman filter paper (No. 1, Whatman Inc., Puscataway, NJ, USA) and then diluted 5-fold. Total sulfhydryl contents in 5-fold diluted supernatant was analyzed. After adding 2 mL of 0.1 M Tris buffer (pH 8.0) and 0.5 mL of 10 mM DTNB in 0.1 M Tris buffer (pH 8.0) to 0.5 mL sample, the mixture was shielded from light and allowed to react for 30 min. Absorbance was then measured spectrophotometrically at 412 nm. A solution containing 0.5 mL of 5% SDS in Tris buffer (pH 8.0), 0.5 mL of 10 mM DTNB, and 2.0 mL of 0.1 M Tris buffer (pH 8.0) was used as reagent blank. Protein content was determined using a standard curve of bovine serum albumin at wavelength of 550 nm through biuret reaction. Sulfhydryl content was computed using the following equations:

$$\begin{array}{l} \text{i})\;\text{Molecular extinction coefficient}=\text{E}412\\ =14,000\;{\text{M}}^{-1}{\text{cm}}^{-1}\\ \text{Thiol concentration }(\text{nM})=\text{OD}\times \text{DF}/0.014\;{\text{nM}}^{-1}{\text{cm}}^{-1}\\ \text{ii})\;\text{Thiol nM}/\text{mg}=\text{Thiol concentration }(\text{nM})/\text{protein concentration }(\text{mg}) \end{array}$$

### Texture profile analysis

Texture profile analysis (TPA) of patties was carried out in triplicates for each treatment using a Texture Analyzer (CT3-1000, Brookfield Engineering Laboratories, Inc., Middelbro, MA, USA). Six samples were cut approximately 25 mm×10 mm (diameter×height) from central section of each patty treatment. Before analysis, samples were allowed to equilibrate at room temperature for 30 min. The setting was: distance = 6.0 mm, pre-test speed = 2.0 mm/s, post-test speed = 8.0 mm/s, head speed = 2.0 mm/s, and force = 5.0 g. Patties of TPA values for springiness, gumminess (kg), cohesiveness, hardness (kg), and chewiness (kg) were determined using methods describe by Bourne [22].

### Sensory evaluation

Sensory evaluation was conducted at Konkuk University, Republic of Korea. Panels were made up of ten members who have experience in meat product sensory evaluation (graduates and faculty, both sexes and age of 23 to 55 years, Konkuk University, Department of Food Science and Biotechnology of Animal Resources). The panels were selected through three preliminary tests to recognize differences among the groups. Cooked patties were refrigerated overnight and allowed to stand at room temperature for 30 min before evaluation. Each sample slice (10×10×10 mm) was served to each panelist, randomly. The panels were asked to evaluate the quality of samples at a day in duplicate (morning and afternoon). Cooked patties were evaluated for flavor (1 = undesirable to 7 = desirable), taste (1 = undesirable to 7 = desirable), texture (1 = tough to 7 = tender), juiciness (1 = dry to 7 = juicy), and overall preference (1 = undesirable to 7 = desirable) using a seven-point hedonic scale.

### Statistical analysis

A randomized block experiment design with five treatments (Control, T1, T2, T3, and T4) and post-treatment (cooked and reheated) was used in triplicate. Effect of AG powder and processing application on each measured parameter was analyzed by two-way analyses of variance using IBM SPSS statistics 24.0. Post-treatment exclusively included fresh patties, cooked, and reheated samples in analysis of color and MRPs. Data are presented as mean±standard error of means. Significant differences between different treatments were analyzed using Tukey test. A p-value of less than 0.05 was considered statistically significant.

## RESULTS AND DISCUSSION

### Physicochemical properties

Proximate composition (moisture, crude protein, crude fat, and ash) of raw patties among the control, T1, T2, T3, and T4 did not show significant differences among groups (proximate composition data of raw patties not shown). As shown in Table 1, moisture (46.49% to 48.06%) of RTE pork patties was not affected by the addition of FG/AG powder. Moisture content was decreased from 50.66% to 43.73% by reheating regardless of the addition of FG/AG powder since the moisture content in samples was dehydrated during the heat process.

A decreasing tendency of pH was detected with increasing AGP concentration (p<0.001, Table 1) and the pH of T1 was significantly higher than that of the control group. Similar to our results, Shin et al [9], found that pH of AG added sausages decreased during storage. Pre- and post-treatment interaction effect of pH was proved (p<0.001) since pH was increased from 6.37 to 6.41 in the control group but decreased in T2 (6.38 to 6.26), T3 (6.28 to 6.17), and T4 (6.21 to 5.96). It has been reported that the heating process for AG can cause acidification [5].

Results of cooking loss of pork patties with added FG/AG powder after roasting and reheating are shown in Table 1. Cooking loss of T4 was significantly higher than that of the control group (p<0.05), whereas cooking loss of T1, T2, or T3 did not show significant difference from that of the control group (p>0.05). According to El-Magoli et al [23], water-holding and fat binding abilities of additive are main factors reducing the cooking loss of meat product. This indicates that water and fat binding capacities of AG powder might be insignificant.

### Color

Results of color (lightness, redness, and yellowness) measurement of fresh pork patties with added FG/AG powder are shown in Table 2. A pre- and post-treatment interaction was observed (p<0.001) for L* (lightness), a* (redness), and b* (yellowness). Lightness of fresh pork patties was not affected by the addition of 1% FG powder (T1). However, the lightness was reduced linearly with increasing level of AGP added at post-treatment (p<0.001). In terms of raw patties, redness value of T1, T2, T3, or T4 was significantly lower than that of the control group. However, redness values of T1, T2, T3, and T4 at post-treatment increased linearly with increasing level of AGP. In addition, the redness of the control group was decreased at post-treatment. These results indicated that the addition of AGP could reduce lightness but increase redness in cooked food with added AGP. This could be due to activation of Maillard reaction accompanied by increased contents of total sugar and amino acids in AG [5,10]. Yellowness was increased linearly with increasing level of AG compared to that of the control group (p<0.001). Regarding the interaction effect of L*, L* reduction of T4 (from 35.63 to 32.50) was less affected than that of other groups after reheating. Moreover, b* reduction of T4 (from 21.52 to 16.82) was more affected than that of other groups after the reheating process. Similarly, Shin et al [9] have reported that b* value of sausage with added black garlic extracts is higher than sausage without addition of black garlic extracts.

### Brown intensity

**Figure 2:**
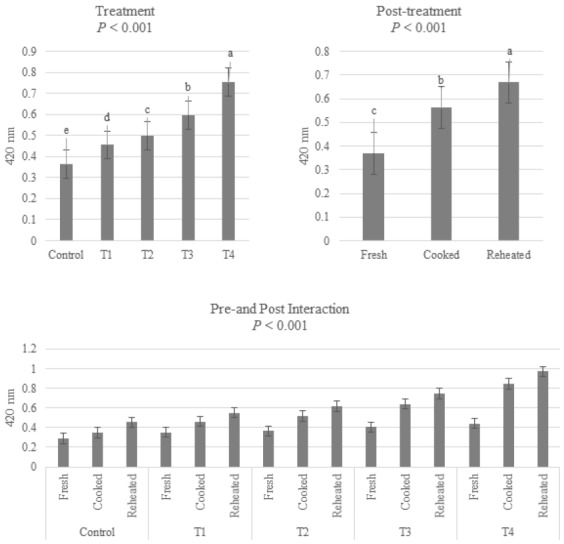
Brown intensity of fresh, cooked and reheated pork patties added fresh/aged garlic powder. Control, pork patties without fresh or aged garlic powder; T1, 1% fresh garlic (w/w), T2, T3, and T4, 0.5%, 1%, and 2% aged garlic (w/w).

The effect of addition of FG or AG on the BI of fresh, cooked, and reheated pork patties is shown in Figure 2. The BI was increased with increasing level of AG (p<0.001). It gradually increased throughout the cooking procedure (p<0.001). The BI at 420 nm of garlic has been considered as Maillard reaction [24], particularly from the point of view of non-enzymatic browning as shown in Figure 1. An increase in Maillard reaction of AGP during heating has been reported (6), showing that BI and sulfur compounds of garlic are increased by ageing time and heating process. Yoo et al [25] have reported that BI of roasted sesame is positively correlated with its antioxidant activity. In addition, pre-treatment and post-treatment had significant effects on BI (p<0.001) since the increase of BI with increasing level of AG was more affected by post-treatments.

### 2-Thiobarbituric acid reactive substances

The TBARS of RTE pork patties with added fresh/AGP did not have interaction effect (p>0.05, Table 1). The TBARS in RTE pork patties was reduced with increasing level of AGP (Control, 1.92; T1, 1.49; T2, 1.20; T3, 0.86; and T4, 0.52 MDA mg/kg meat, respectively). The TBARS was increased from 1.03 to 1.37 MDA mg/kg meat after the reheating process (p< 0.001). Despite the addition of the same concentration (T1, 1% FG and T3, 1% AG), T3 inhibited lipid oxidation more efficiently than T1. Tarladgis et al [20] have found that the perception of rancidity flavor is related to TBARS (from 0.5 to 1.0 in pork). The effect of the addition of AGP on inhibition of lipid oxidation was verified at both process (cooking and storing/reheating). Antioxidant effect of aged black garlic has been reported in previous studies [8,26]. In particular, MRPs of AG in the reheated samples could be generated during the heating process, resulting in the inhibition of lipid oxidation [19,25]. Garlic has stable antioxidant ability after boiling or frying treatment, although its antioxidant ability is lower than FG [27]. In addition, the antioxidant ability of AG increased with increasing heat temperature (from 40°C to 85°C) during manufacturing [6].

### Sulfhydryl content

Sulfhydryl contents of RTE pork patties with added FG/AG powder and post-treatment are shown in Table 1. Sulfhydryl content did not show an interaction effect between pre- and post-treatments (p>0.05). Sulfhydryl content in T3 or T4 (8.45 and 9.98 mM/kg protein, respectively) was considerably higher than that of the control group (6.68 mM/kg protein, p< 0.001). However, sulfhydryl content of the control group did not differ from that of T1 or T2 (p>0.05). Sulfhydryl content was decreased from 9.15 to 7.24 mM/kg protein after reheating regardless of pre-treatment. Botsoglou et al [28] have demonstrated that natural antioxidant extracts can inhibit the reduction of sulfhydryl contents of cooked patties during refrigerated storage. These results indicated that the addition of 1.0% to 2.0% AG powder inhibited the reduction of sulfhydryl contents in cooked and reheated pork patties compared to non-additives or addition of FG addition at the same concentration. According to Kim et al [29], AG contains higher contents of S-allyl-L-cysteine and diallyl-disulfide (522.51±1.19 and 8.71±0.45 μg/g sample, respectively) compared to FG (245.35±1.35 and 0.275±0.15 μg/g sample, respectively). The inhibition of the reduction of sulfhydryl content in FG and AG during the processing could be related to their sulfur-containing antioxidants such as allyl cysteine and allyl disulfide [1].

### Texture characteristics

Texture profiles of cooked/reheated pork patties with added FG/AG powder are shown in Table 3. Pork patties with added AGP showed higher values for gumminess, hardness, and chewiness than those from the control group (p<0.05). The springiness and cohesiveness did not differ among treatment groups (p>0.05). Reheating process significantly increased gumminess, hardness, and chewiness but decreased the springiness. Similar changes in texture profiles (reduction of springiness but increases of gumminess, hardness, and chewiness) by reheating process have been reported for chicken patties with added lotus leaf powder after storage in a refrigerator [30]. In addition, pre- and post-treatment interaction effect of gumminess, hardness, and chewiness occurred (p< 0.001) since patties with the highest concentration of AGP (T4) was more affected by the post-treatment compared to control or T1 group. Such changes in texture profiles indicate that polymerization of sulfhydryl-disulfide interchange reaction might have induced protein aggregation by heating due to plentiful thiol groups in AG [29]. This is consistent with results of Andic et al [31] showing that beef patties with added whey powder contain plentiful thiol groups, showing increased hardness and chewiness, in agreement with Ulu [32].

### Sensory characteristics

The effect of fresh/pork addition on texture and juiciness of pork patties results was found to be insignificant (Table 4). However, significant inclining trend score was found in color, flavor, taste, and overall preference of pork patties with increasing levels of AGP (p<0.05). Significant effects of post-treatment on texture and overall preference were observed. The effect of pre-and post-treatment interaction on sensory characteristics was insignificant (p>0.05).

## CONCLUSION

The AG added at concentrations of 0.5%, 1.0%, and 2% affected physicochemical, texture, and sensory characteristics as well as lipid/protein oxidation of RTE pork patties. In terms of oxidative stability, AG at concentration of 1.0 and 2.0 g/100 g patties showed antioxidant effect on lipid and protein oxidation. In addition, color of patties with added AG at 2.0 g/100 g might be acceptable because of Maillard reaction. Although significant difference in flavor, taste, or overall acceptability among patties with either added FG or AG was not observed, however, they showed increasing tendencies with an increasing level of AG. However, high concentrations (1.0 and 2.0 g/ 100 g patties) of AG showed negative effects on cooking loss and texture characteristics. Therefore, addition of AG to meat products might be able to extend their shelf-life and improve their sensory characteristics. However, further studies are needed to understand the relationship between AG and Maillard reaction in meat products as well as functionalities of MRPs from AG.

## Figures and Tables

**Table 1:** Moisture, cooking loss (%), pH, TBARS, and sulfhydryl contents of ready-to eat pork patties with added fresh and aged garlic

Items		Moisture (%)	pH	Cooking loss (%)	TBARS1)	Sulfhydryl2)
Treatment						
Control3)		46.57	6.39^b^	20.66^b^	1.92	6.68
T13)		47.54	6.41^a^	21.85^ab^	1.49	7.70
T23)		47.32	6.32^c^	21.87^ab^	1.2	8.15
T33)		48.06	6.23^d^	21.44^b^	0.86	8.45
T43)		46.49	6.09^e^	22.32^a^	0.52	9.98
SEM		0.651	0.006	0.395	0.105	0.388
p-value		0.385	<0.001	<0.05	<0.001	<0.001
Post-treatment						
Cooked		50.66	6.33	27.37	1.03	9.15
Reheated		43.73	6.24	15.88	1.37	7.24
SEM		0.411	0.004	0.25	0.067	0.245
p-value		<0.001	<0.001	<0.001	<0.001	<0.001
Treatment×post-treatment						
Control	×Cooked	50.49	6.37	25.72	1.49	7.74
	×Reheated	42.64	6.41	15.60	2.36	5.62
T1	×Cooked	50.33	6.43	27.43	1.31	8.81
	×Reheated	44.76	6.40	16.26	1.68	6.60
T2	×Cooked	50.79	6.38	27.46	1.15	9.32
	×Reheated	43.85	6.26	16.28	1.25	6.98
T3	×Cooked	51.53	6.28	27.29	0.81	9.86
	×Reheated	44.60	6.17	15.59	0.91	7.04
T4	×Cooked	50.16	6.21	28.97	0.41	10.01
	×Reheated	42.82	5.96	15.66	0.64	9.95
SEM		0.920	0.008	0.542	0.149	0.549
p-value		0.792	<0.001	0.069	0.062	0.126

SEM, standard error of means.

1) Malondialdehyde mg/kg meat.

2) nM/mg protein.

3) Control, pork patties without fresh or aged garlic powder; T1, 1% fresh garlic (w/w); T2, T3, and T4, 0.5%, 1%, and 2% aged garlic (w/w).

a–e Means within a column followed by different superscripts are significantly different (p<0.05).

**Table 2:** Color of fresh and ready-to eat pork patties with added fresh and aged garlic

Items		L*	a*	b*
Treatment				
Control1)		52.29^a^	7.71^d^	15.91^d^
T11)		49.44^b^	7.71^d^	19.45^c^
T21)		45.64^c^	8.31^c^	20.07^b^
T31)		42.50^d^	9.16^b^	21.27^a^
T41)		37.76^e^	9.68^a^	19.44^b^
SEM		0.22	0.09	0.13
p-value		<0.001	<0.001	<0.001
Post-treatment				
Fresh		52.41^a^	8.97^a^	16.17^c^
Cooked		44.80^b^	8.32^b^	21.86^a^
Reheated		39.34^c^	8.24^b^	19.65^b^
SEM		0.15	0.06	0.09
p-value		<0.001	<0.001	<0.001
Treatment×post-treatment				
Control	×Fresh	58.69	11.31	11.42
	×Cooked	53.05	5.78	18.44
	×Reheated	45.10	6.02	17.87
T1	×Fresh	57.76	8.41	13.63
	×Cooked	48.02	7.51	23.15
	×Reheated	42.51	7.20	21.56
T2	×Fresh	51.75	8.20	16.51
	×Cooked	45.76	8.36	22.93
	×Reheated	39.40	8.35	20.78
T3	×Fresh	48.72	8.22	19.28
	×Cooked	41.56	9.61	23.26
	×Reheated	37.20	9.63	21.25
T4	×Fresh	45.14	8.69	19.99
	×Cooked	35.63	10.34	21.52
	×Reheated	32.50	10.00	16.81
SEM		0.39	0.16	0.22
p-value		<0.001	<0.001	<0.001

SEM, standard error of means.

1) Control, pork patties without fresh or aged garlic powder; T1, 1% fresh garlic (w/w); T2, T3, and T4, 0.5%, 1%, and 2% aged garlic (w/w).

a–e Means within a column followed by different superscripts are significantly different (p<0.05).

**Table 3:** Texture profiles of ready-to eat pork patties with added fresh and aged garlic

Items		Springiness (mm)	Gumminess (kg)	Cohesiveness	Hardness (kg)	Chewiness (kg)
Treatment						
Control1)		0.83	3.13^c^	0.48	6.52^d^	2.58^b^
T11)		0.83	2.46^d^	0.47	5.25^c^	2.03^c^
T21)		0.83	3.31^bc^	0.48	6.85^bc^	2.76^ab^
T31)		0.83	3.49^b^	0.49	7.32^ab^	2.89^a^
T41)		0.82	4.02^a^	0.47	8.04^a^	3.13^a^
SEM		0.006	0.115	0.010	0.240	0.102
p-value		0.845	<0.001	0.629	<0.001	<0.001
Post-treatment						
Cooked		0.841	2.80	0.49	5.50	2.29
Reheated		0.815	3.76	0.47	8.09	3.07
SEM		0.004	0.070	0.007	0.478	0.062
p-value		<0.001	<0.001	0.006	<0.001	<0.001
Treatment× post-treatment						
Control	×Cooked	0.84	2.51	0.49	5.11	2.10
	×Reheated	0.82	3.75	0.47	7.94	3.06
T1	×Cooked	0.84	2.18	0.48	4.34	1.84
	×Reheated	0.81	2.75	0.45	6.15	2.23
T2	×Cooked	0.85	3.04	0.50	6.12	2.57
	×Reheated	0.82	3.58	0.46	7.58	2.94
T3	×Cooked	0.4	3.04	0.50	6.06	2.56
	×Reheated	0.82	3.94	0.47	8.58	3.22
T4	×Cooked	0.84	3.25	0.48	5.89	2.36
	×Reheated	0.81	4.80	0.47	10.20	3.91
SEM		0.008	0.158	0.015	0.330	0.140
p-value		0.940	<0.05	0.857	<0.001	<0.001

SEM, standard error of means.

1) Control, pork patties without fresh or aged garlic powder; T1, 1% fresh garlic (w/w); T2, T3, and T4, 0.5%, 1%, and 2% aged garlic (w/w).

a–e Mean within a column followed by different superscripts are significantly different (p<0.05).

**Table 4:** Sensory evaluation of ready-to eat pork patties with added fresh and aged garlic

Items		Color	Flavor	Taste	Texture	Juiciness	Overall preference
Treatment							
Control1)		2.50^c^	2.85^b^	2.95^b^	4.20	4.25	3.35^b^
T11)		3.20^c^	4.25^a^	4.95^a^	4.90	4.45	4.70^a^
T21)		4.35^b^	4.45^a^	4.60^a^	4.20	4.05	4.70^a^
T31)		5.20^ab^	5.00^a^	5.20^a^	4.25	4.10	5.15^a^
T41)		5.60^a^	5.45^a^	5.20^a^	4.55	4.30	4.95^a^
SEM		0.273	0.332	0.310	0.332	0.342	0.311
p-value		<0.001	<0.001	<0.001	0.501	0.926	<0.001
Post-treatment							
Cooked		4.12	4.12	4.36	4.06	4.14	4.26
Reheated		4.22	4.68	4.80	4.78	4.32	4.88
SEM		0.173	0.210	0.196	0.210	0.216	0.197
p-value		0.683	0.063	0.116	<0.05	0.557	<0.05
Treatment×post-treatment							
Control	× Cooked	2.70	2.70	2.70	3.70	3.80	3.10
	×Reheated	3.00	3.00	3.20	4.70	4.70	3.60
T1	×Cooked	3.80	3.80	4.30	4.30	4.10	4.20
	×Reheated	4.70	4.70	5.60	5.50	4.80	5.20
T2	×Cooked	4.10	4.10	4.70	4.10	4.60	4.40
	×Reheated	4.80	4.80	4.50	4.30	3.50	5.00
T3	×Cooked	4.90	4.90	5.20	4.00	4.10	5.10
	×Reheated	5.10	5.10	5.20	4.50	4.10	5.20
T4	×Cooked	5.10	5.10	4.90	4.20	4.10	4.50
	×Reheated	5.80	5.80	5.50	4.90	4.50	5.40
SEM		0.386	0.470	0.439	0.470	0.483	0.440
p-value		0.830	0.938	0.473	0.840	0.261	0.859

SEM, standard error of means.

1) Control, pork patties without fresh or aged garlic powder; T1, 1% fresh garlic (w/w); T2, T3, and T4, 0.5%, 1%, and 2% aged garlic (w/w).

a–e Means within a column followed by different superscripts are significantly different (p<0.05).
